# A Comparison of Averaged and Full Models to Study the Third-Body Perturbation

**DOI:** 10.1155/2013/136528

**Published:** 2013-11-03

**Authors:** Carlos Renato Huaura Solórzano, Antonio Fernando Bertachini de Almeida Prado

**Affiliations:** ^1^Federal University of ABC (UFABC), Rua Santa Adélia, 166 Bairro Bangu, 09.210-170 Santo André, SP, Brazil; ^2^National Institute of Research Space (INPE), Avenida dos Astronautas, 1758 Jd da Granja, 12227-010 São José dos Campos, SP, Brazil

## Abstract

The effects of a third-body travelling in a circular orbit around a main body on a massless satellite that is orbiting the same main body are studied under two averaged models, single and double, where expansions of the disturbing function are made, and the full restricted circular three-body problem. The goal is to compare the behavior of these two averaged models against the full problem for long-term effects, in order to have some knowledge of their differences. The single averaged model eliminates the terms due to the short period of the spacecraft. The double average is taken over the mean motion of the satellite and the mean motion of the disturbing body, so removing both short period terms. As an example of the methods, an artificial satellite around the Earth perturbed by the Moon is used. A detailed study of the effects of different initial conditions in the orbit of the spacecraft is made.

## 1. Introduction

The effects of the gravitational attractions of the Sun and the Moon in the orbits of an artificial satellite of the Earth have been studied in several papers. Kozai [1] writes Lagrange's planetary equations using the disturbing function due to the Sun and the Moon, including both secular and long periodic terms, but he only gives explicit expressions for the secular terms. Blitzer [2] ignores the specialized techniques of celestial mechanics and obtains estimates for the perturbations using methods of classical mechanics. Again, only secular terms are included, and it shows that the main effect is a precession of the orbital plane around the pole of the ecliptic. Musen [3] shows two systems of formulas for the determination of the long periodic perturbations. The first system uses the theory originally developed by Gauss for a numerical treatment of the very long periodic effects in planetary motion, and the second method is based on the development of the disturbing function in terms of the Legendre polynomials, and it finds long periodic terms and its influence on the stability of the orbit. Through linear analyses, Frick and Garber [4] show that the result of the lunisolar attraction is a change in the orbital plane with small oscillations. Kaula [5] derived general terms from the disturbing function for the lunisolar disturbance using equatorial elements for the Moon and the Sun. Cook [6] studied the perturbations due to solely a third body from Lagrange's planetary equations by integrating them over one revolution of the satellite. The rates of change of the orbital elements averaged over one revolution are then written and all first order terms (secular and long period) are retained in the analysis. The theory is limited to satellites whose semimajor axis does not exceed one-tenth of the distance from the Earth to the Moon.

The effects of the Sun and the Moon on a near-equatorial synchronous satellite under the influence of the gravitational fields of an oblate Earth, the Sun, and the Moon are studied by Zee [7]. The disturbing function for the perturbations due to the Moon using ecliptic elements for the Moon, and equatorial elements for the satellite are also considered in the literature. Secular, long period, and short period perturbations are computed, with the expressions kept in closed forms in both inclination and eccentricity of the satellite. Alternative expressions for short period perturbations of high altitude satellites are also given by Giacaglia [8], assuming small values of the eccentricity. In another work, Kozai [9] developed an alternative method for the calculation of the lunisolar disturbances. The disturbing function was expressed in terms of the orbital elements of the satellite and the geocentric coordinates of the Sun and the Moon. The secular and the long period terms are derived by numerical integration, and the short period terms are derived analytically. 

The singularities from the geopotential expansion and its derivates are removed, but a mixed set of orbital elements is allowed to remain in the expansions (see references [10–12]). However, the latter development retains much of the original inclination and eccentricity functions, allowing their calculation by existing recursion relations. Nacozy and Dallas [13] removed singularities from the geopotential and its derivates for zero eccentricity and inclination. The geopotential expansion is obtained entirely in terms of nonsingular orbital elements.

Using the Hamiltonian formed by a combination of the declination and the right ascension of the satellite, the Moon, and the Sun, Hough [14] studied the periodic perigee motion for orbits near the critical inclinations 63.4° and 116.6°. The theory predicts the existence of larger maximum fluctuations in eccentricity and faster oscillations near stable equilibrium points. The lunisolar effects in a geosynchronous artificial satellite orbiting near the critical inclination is studied by Delhaise and Morbidelli [15], by analyzing each harmonic formed by a combination of the satellite and the longitude of the node of the Moon. This study demonstrates that the dynamics induced by these harmonics does not show resonance phenomena. Breiter [16] studies the third-body effects in the resonance of the apsides for satellites in low altitude, determining the resonant eccentricities between the secular motion of a satellite in terrestrial orbit and the longitudes of the Moon and the Sun. This study was made in a Hamiltonian form. References [17–22] study the long-term effects of the gravitational perturbations caused by a distant body. The third-body disturbing function is truncated in a higher degree.

All these works present basic contributions in the area and possess an analytical approach, rich in derivations of equations. In the present work, a semianalytic and a numerical approach are used, with the goal of complementing the existing literature. Our main goal is to compare the single and the double averaged models with the full restricted circular three-body problem. During this comparison several aspects of the orbits selected are studied, with particular attention to the effects of the initial orbit of the spacecraft in the evolution of the Keplerian elements due to the third-body perturbation.

## 2. Mathematical Models

**Figure 1:**
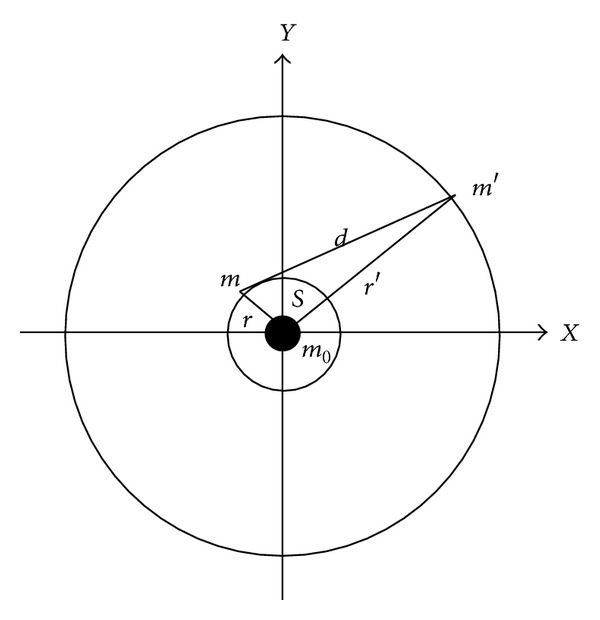
Third-body perturbation.

The model used in all the methods used in the present study is similar to the planar restricted three-body problem. There are three bodies involved in the dynamics: one body with mass *m*_0_ fixed in the origin of the reference system, a second massless body in a three-dimensional orbit around *m*_0_, and a third body in a circular orbit around *m*_0_ that disturbs the orbit of the second body (see Figure 1). So, the motion of the spacecraft (the massless body) is three-dimensional, with its orbital elements perturbed only by the third body. Under those assumptions, it is possible to study this problem numerically using the full dynamics or using averaged models, which have the goal of removing short period terms in order to study only the long-term behavior of the spacecraft. The averaged models can be divided in two main groups: the single averaged models, which eliminate only the periodic terms of the satellite; and the double averaged models, which eliminate the short periodic terms due to the satellite and the disturbing body. Both averaged models are usually based in series expansions, so it is also necessary to choose a limit for the terms for the truncation of the expansions. Details about the specific models considered in the present paper are shown below. 

### 2.1. Single Averaged Model

In this situation, the average is performed with respect only to the true anomaly of the spacecraft (*f*). The short periodic terms due to the disturbing body are kept in the dynamics. The disturbing function is then expanded in Legendre polynomials. The approach used here follows the solutions presented in reference [17]. 

The main body *m*_0_ is fixed in the center of the reference system *X*-*Y*. The perturbing body *m*′ is in a circular orbit with semimajor axis *a*′ and mean motion *n*′. The spacecraft is in a three-dimensional orbit, with orbital elements: *a, e, i, *ω*,* and *Ω*, and mean motion *n *(see Figure 1). In this situation, the disturbing potential that the spacecraft has from the action of the perturbing body is given by using the expansion in Legendre polynomials and assuming that *r*′≫ *r *[17]:

(1) R=μ′G(m0+m′)r2+r′2−2rr′cos⁡⁡(S)=μ′(m0+m′)r′∑n=2∞(rr′)nPncos⁡⁡(S).

The several parts of the disturbing potential due to the expansion in Legendre polynomials are averaged over the short period of the satellite. The definition for average used in the present paper is

(2) $$\begin{array}{c} \langle G\rangle =\frac{1}{2\pi }\overset{2\pi }{\underset{0}{\int }}GdM, \end{array}$$

where *M* is the mean anomaly of the satellite and *M*′ is the mean anomaly of the perturbing body. The results obtained here are valid for the special case of circular orbits for the perturbing body and with the initial mean anomaly of the perturbing body equal to zero. 

After the process of averaging, the equations of motion of the spacecraft are obtained from the Lagrange's planetary equations that are written as a function of the derivatives of the disturbing function [23]. It is noticed that the semimajor axis always remains constant, because after the averaging, the disturbing function does not depend on the variable *M*_0_.

Lagrange's planetary equations have the disadvantage of showing singularities in the eccentricity and/or inclination for circular and/or planar orbits, respectively. The presence of the eccentricity and the inclination in the denominator causes these singularities, but this fact is mathematical and not physical. The longitude of the ascending node is also not defined for zero inclination. The circular orbits have the argument of perigee indefinite and the equatorial orbits show the same behavior for the longitude of the ascending node. It is possible to express these equations in terms of the nonsingular elements. We can use the equations of motion determined by Giacaglia [10] in nonsingular elements considering that *I* ≠ *π* and *e* < 1.

### 2.2. Double Averaged Model

The double averaged model considers the averages over the mean motion of the satellite and the mean motion of the disturbing body. The second average is made with respect to the disturbing body to eliminate *M*′. It is necessary to hold the Keplerian elements of the spacecraft constant during the process of averaging. This is possible due to the hierarchy of timescales, since the period of the satellite is much smaller than the period of the slow oscillations of the orbital elements [18]. The final equations used in the present paper are available in reference [18] and they are not repeated here.

### 2.3. The Restricted Three-Body Problem

The motion of a small particle with negligible mass moving under the gravitational influence of two masses *m*_1_ and *m*_2_, without any type of average or truncations, is now considered. It is assumed that the two masses are in circular orbits around their center of mass and that they generate a force that governs the motion of the particle, although the particle does not affect the motion of the two primaries. The equations of motion in the synodic reference system are [24, 25]

(3) $$\begin{array}{c} \ddot{x}-2\dot{y}-x=-\mu_{1}\frac{x+\mu_{2}}{r_{1}^{3}}-\mu_{2}\frac{x-\mu_{1}}{r_{2}^{3}},\\ \ddot{y}+2\dot{x}-y=-y\left(\frac{\mu_{1}}{r_{1}^{3}}+\frac{\mu_{2}}{r_{2}^{3}}\right), \end{array}$$

where

(4) $$\begin{array}{c} r_{1}^{2}={\left(x+\mu_{2}\right)}^{2}+y^{2},\\ r_{2}^{2}={\left(x-\mu_{1}\right)}^{2}+y^{2}. \end{array}$$

## 3. The Critical Inclination Problem

Many authors studied the motion of satellites that are close to the Earth with inclination near the critical value [26–29], which is a value for the inclination between the orbital planes of the perturbing and the perturbed bodies, such that any near-circular orbit with inclination below this value remains near circular. This critical angle is 39.231 deg. However, some of these authors considered only terms that are of the order of the square of *J*_2_ in the equations of motion. Izsak [30] shows that continuations from the ordinary treatment of the libration to the higher approximations would break down and, in the case of small eccentricities, some libration of a peculiar kind would occur if the next order of magnitude term is included. Solutions for the problem, not only for the case of small eccentricities but also for the case including terms up to the third order of *J*_2_ are shown by Aoki [31].

For the case of the third-body perturbation, Kozai [32] studied the secular behavior of the satellite, which can be divided into two cases: when the argument of the pericenter circulates and when it librates. According to this simplified theory, for all satellites with inclination below 39.2° (or greater than 140.8°), *ω* circulates from 0 to 2*π*, while for orbits with higher inclinations (39.2° < *i* < 140.8°) *ω* either circulates or librates around 90° or 270°. The last case is what is usually referred to as the Kozai resonance. The first natural satellites observed to be in the Kozai resonance were Kiviuq and Ijirak [33–36], which are satellites of Saturn.

### 3.1. Direct Orbits

**Figure 2:**
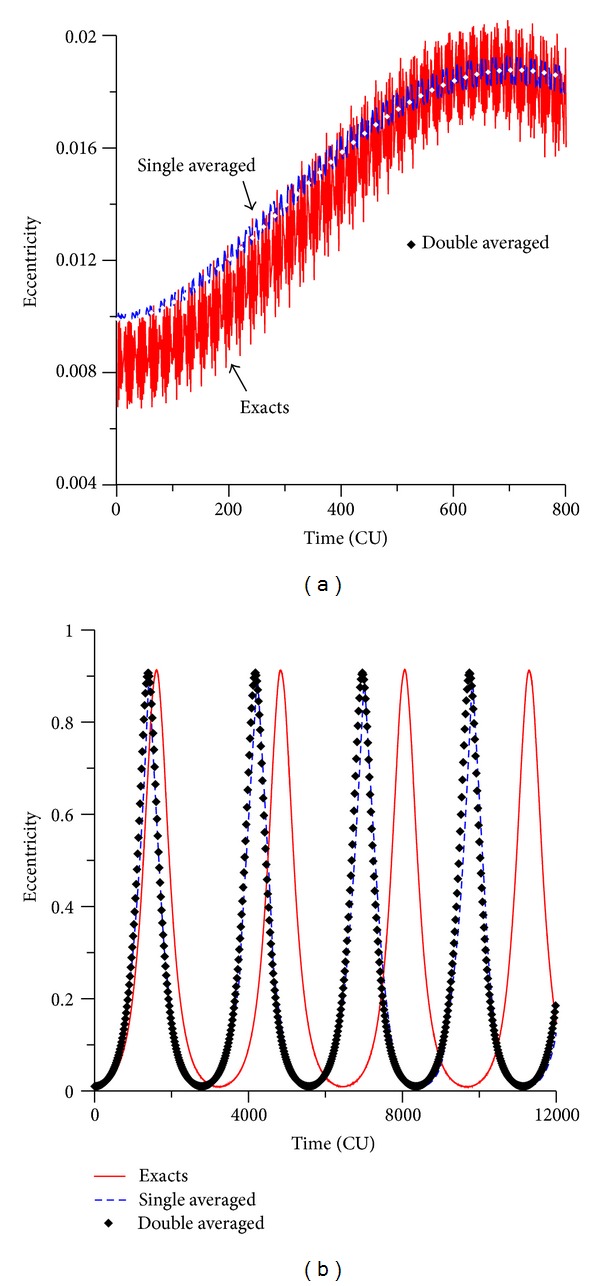
Evolution of the eccentricity for values of the inclination of 30° (a) and 70° (b). Initial conditions are *a* = 0.341 canonical units, *e* = 0.01, *ω* = 0, and Ω = 0.

The numerical simulations made in the present paper started by studying direct orbits. Looking for the behavior of the orbital elements for values of the initial inclination above and below the critical value, it is visible that the inclination oscillates with varied amplitude.  Figure 2(a) shows the behavior of the eccentricity for an initial inclination of 30 deg. It is visible the existence of small variations in the amplitudes. This is important to keep the stability of the orbits. For values of the initial inclination above the critical one (see Figure 2(b), which considers an initial inclination of  70 deg), the oscillations of the eccentricity has high amplitudes. It is shown that near-circular orbits can reach eccentricities with values up to 0.9.

Since the main goal of the present research is to compare the three methods already mentioned to solve this problem, both averaged problems (single and double), and the full model, the results obtained by the three models are shown in Figure 2. The exact model (restricted circular three-body problem) shows results that comprise a region full of values, due to the short periodic terms of the potential due to the spacecraft and the perturbing body. On the other side, as expected, the averaged models show curves composed by fewer points, resulting from the filtration of the short periodic terms. This is visible and can be quantified in Figure 2(a) because the amplitudes of variations are smaller and the scale of the figure allows the clear identification of the three models. A quick inspection can show the relative amplitudes of oscillations from the wide band of the results for the full model (amplitude around 0.003) to the very thin band generated by the double averaged model (just points, with zero amplitude), passing by the intermediate thickness of the single averaged model (amplitude smaller than 0.001). In this situation, a confirmation and a quantification of the expected behaviors are made. Figure 2(b) has more interesting results. The oscillations have larger amplitudes, since the inclination is above the critical angle. The scale of the plot does not allow the visibility of the thickness of the region of results, so the models look the same regarding this aspect, although detailed measurements show that the differences in the thickness of the bands of results still exist. It is also clear that both averaged models have similar behaviors. Compared to the exact model, it is visible that both averaged models have a shorter period for the oscillations, since there is a reduction in the time interval of the peak of the oscillations, which means that the dynamics generated by the truncated methods are faster than the real system. So, the effect of the average process and the consequent truncation is to accelerate the oscillations, and both averaged models have similar, but not exactly identical, reductions of speed. The double averaged model is a little bit faster in those reductions. This fact is also observed in Domingos et al. [37], which studied this problem considering an elliptic orbit for the disturbing body but considering the expansions only up to the second order.

**Figure 3:**
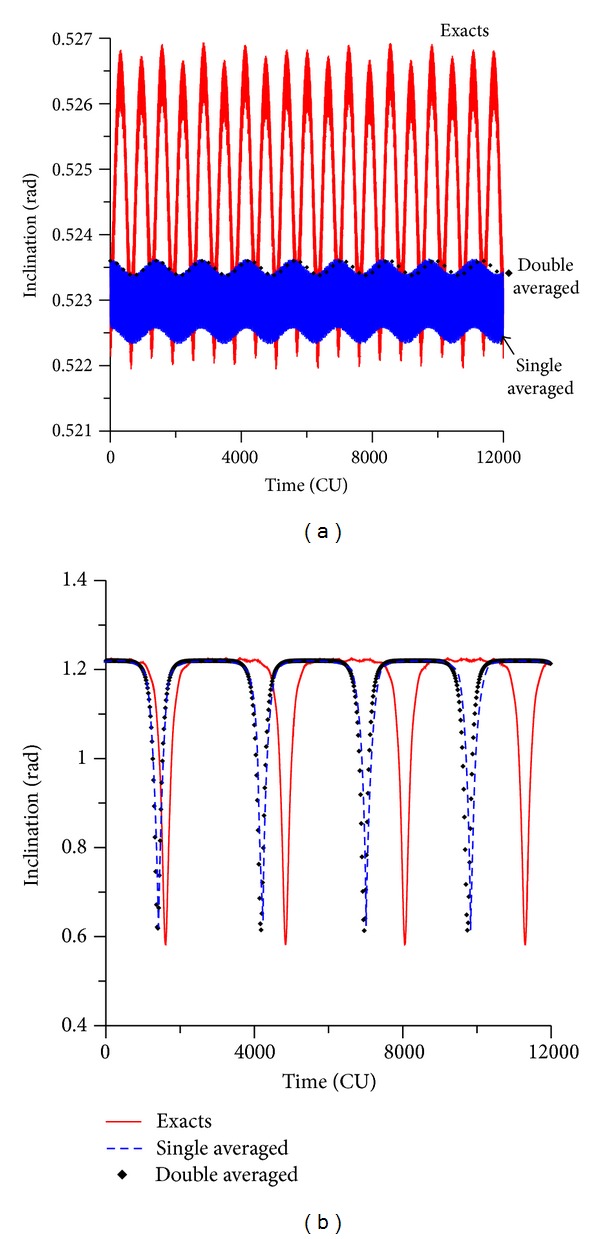
Evolution of the inclination for values of the initial inclination of 30° and 70°. Initial conditions are *a* = 0.341 canonical units, *e* = 0.01, *ω* = 0, and Ω = 0.

The evolution of the inclination shows similar results. For an initial value for the inclination of 30 deg, the exact model shows an amplitude of variation of the order of 0.005 rad (see Figure 3(a)). The single model has oscillations with smaller amplitudes, in the order of 0.001 rad. The double averaged model oscillates around the exact value of the inclination, with near zero amplitude. For the cases of initial inclinations above the critical value (see Figure 3(b) for the inclination of 70 deg), the inclination starts at the initial value, decreases to the critical value, and then it returns again to its original value. All the models make the same correct prediction, but once again the averaged models have results that are very similar to each other, but that predicts an oscillation with smaller period compared with the full model. So, the averaged process and the truncation of the perturbing function have the same effects noticed in the behavior of the eccentricity of accelerating the dynamics.

### 3.2. Retrograde Orbits

**Figure 4:**
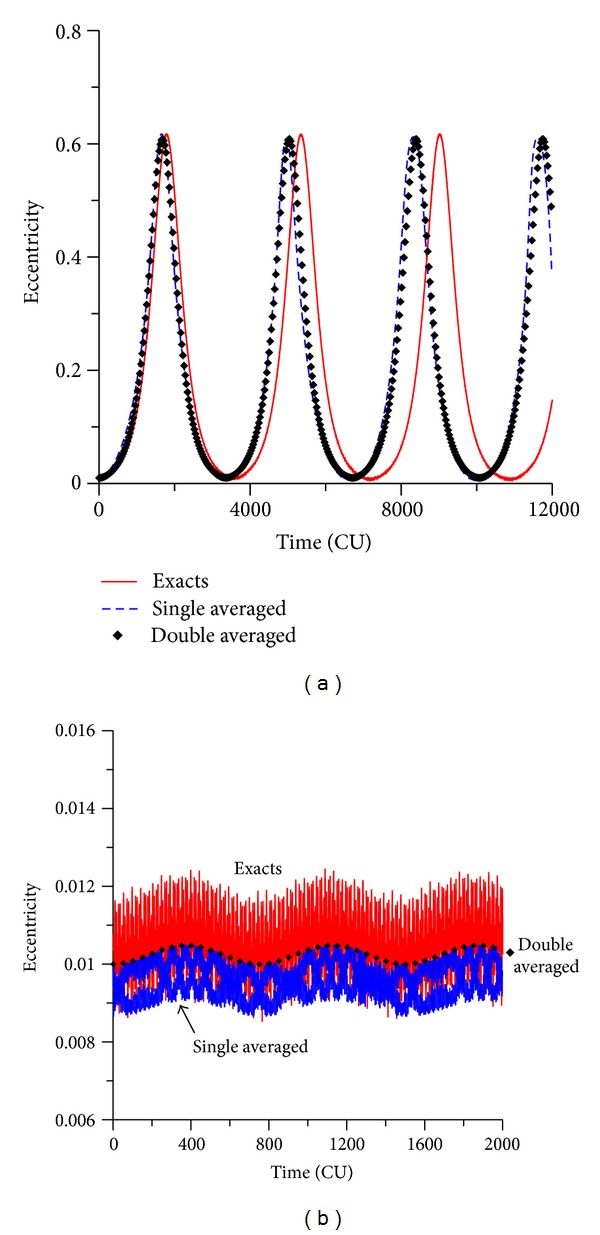
Evolution of the eccentricity for values of the inclination of 130° (a) and 170° (b). The initial conditions are *a* = 0.341 canonical units, *e* = 0.01, *M*_0_(0) = 0, *ω* = 0, Ω = 0.

To make a more complete study, the case of retrograde orbits is considered now. The plots for the evolution of the orbital elements are shown for initial inclinations of 130 deg and 170 deg. It is visible that the evolutions of the inclination for retrograde orbits show a behavior that looks like a mirror image of the results for the direct orbits. For the initial inclination of 130 deg (Figure 4(a)), which is below the critical value for retrograde orbits, the eccentricity reaches high values. The near circular orbits become very elliptic. Comparing the models, it is visible that the same displacement between the averaged models and the exact model noticed for direct orbits happens again. Both averaged models have results that follow each other but that predicts oscillations that have a period shorter than the one given by the full model. For values of the inclination of 170 deg, that is, now above the critical value, the eccentricity has small oscillations, and it is visible the differences in the amplitudes of the three methods used. The exact model has amplitude of oscillation of 0.002, the single averaged model of 0.001, and the double averaged model has zero amplitude of oscillation.

**Figure 5:**
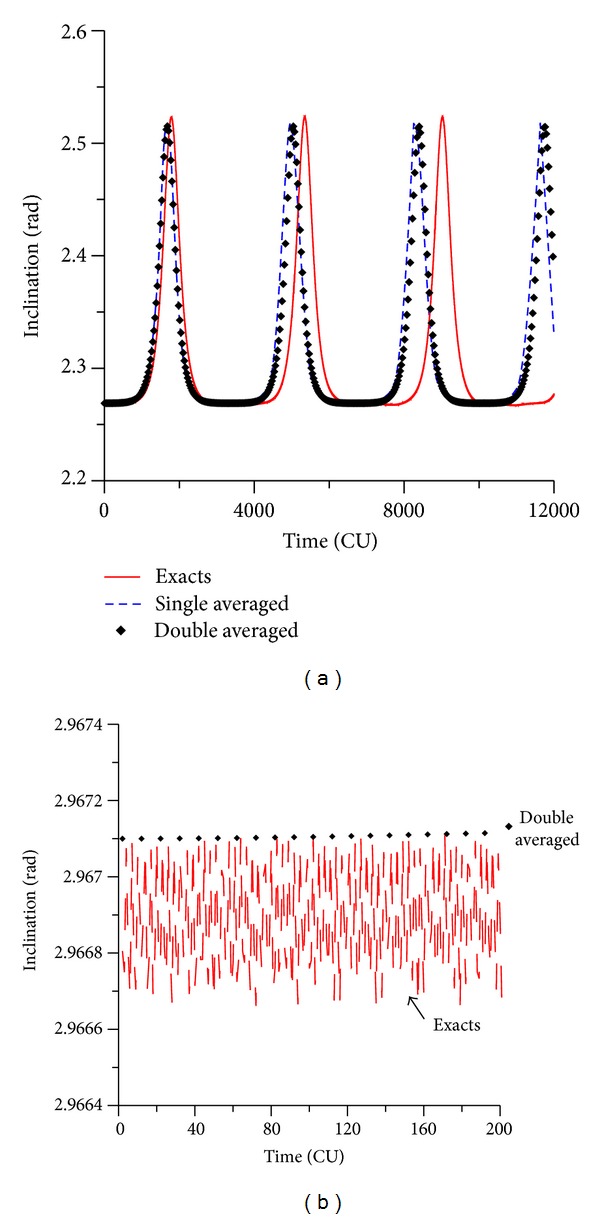
Evolution of the inclination for values of the inclination of 130° and 170°. Initial conditions are *a* = 0.341 canonical units, *e*(0) = 0.01, *M*_0_(0) = 0, *ω*(0) = 0, and Ω(0) = 0. The results for the single and double averaged models are the same.

Considering now the behavior of the inclination, the results for situations where the initial inclination is below the supplement of the critical angle have the same characteristic behavior shown for direct orbits with inclination above the critical angle. The inclination starts with an initial value, increase fast, and, when it reaches the supplement of the critical angle, it reverses the rate of change and starts to decrease until the initial inclination value is reached again. Then, the motion starts again and the inclination keeps oscillating between the initial value and the supplement of the critical angle (Figure 5(a)). The difference from the direct orbits is that now the critical angle is higher than the initial value and the figures have a “U” form, that is, inverted when compared to the direct orbits. In a similar form, the averaged models have the same behavior, but they both also predict oscillations with shorter period when compared to the predictions made by the full model. The small oscillations are visible again for the initial inclination of 170 deg (Figure 5(b)), since the long-term variations in the inclination are smaller and the scale of the plots allows the visualization of the short periodic oscillations. The full model has an amplitude of variation of 0.0004 deg, and the filtering of the short periodic terms of both averaged models causes a near zero amplitude of variation. 

### 3.3. Equatorial and Circular Orbits

Orbits that are equatorial and circular at the beginning of the motion have results which differences are very small between both averaged models. Nonsingular elements were used for the single averaged version, and this model was expanded to the second order in eccentricity and inclination, for this particular case of initial equatorial and/or circular orbits. The effects of the perturbations of the Moon on high-altitude Earth satellites were studied. The behavior of the eccentricity is to stay constant, with some regions where it suffers small variations. The inclination also has the same behavior of staying constant. Since those models show trivial results, only the simulations for the exact model are shown here, because they show the general behavior and the short period oscillations. 

**Figure 6:**
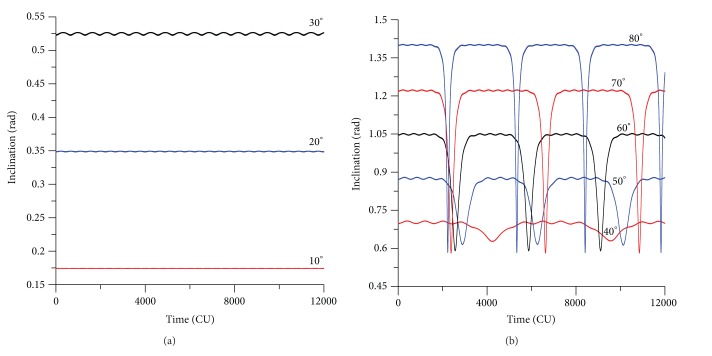
Evolution of the inclination for circular and direct orbits (exact model). The initial conditions are *a* = 0.341 canonical units, *e*(0) = 0, *M*_0_(0) = 0, *ω*(0) = 0, and Ω(0) = 0.

**Figure 7:**
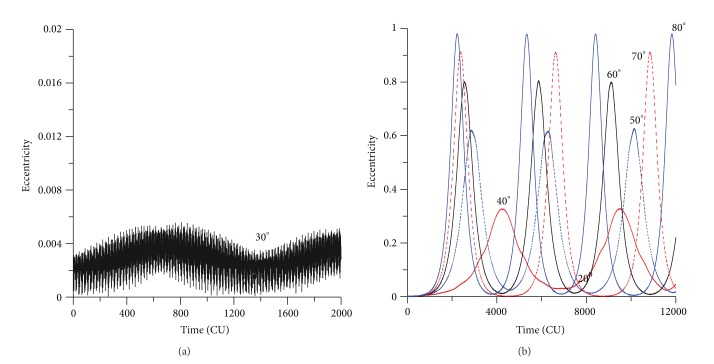
Evolution of the eccentricity for circular and direct orbits (exact model). The initial conditions are *a* = 0.341 canonical units, *e*(0) = 0, *ω*(0) = 0, and Ω(0) = 0.

The inclination and eccentricity for circular and direct orbits have the same behavior shown earlier (see Figures 6 and 7). For values of the initial inclination below the critical value, the eccentricity and inclination suffer small changes (Figures 6(a) and 7(a)), but for values of the inclination above the critical value, the eccentricity and the inclination suffer dramatic changes (Figures 6(b) and 7(b)). The contribution of those two figures is to show the effects of keeping the short periodic terms of the spacecraft and the perturbing body. Note that the behavior is similar to the predictions of the averaged models, but now there are small amplitude fluctuations in the lines due to those terms. Those figures represent the real motion of the spacecraft, without any average or truncations.

**Figure 8:**
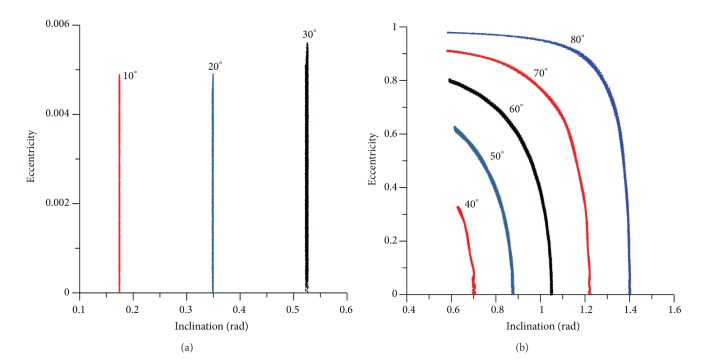
Plots of inclination versus eccentricity for circular and direct orbits (exact model). The initial conditions are *a* = 0.341 canonical units, *e*(0) = 0, *M*_0_(0) = 0, *ω*(0) = 0, and Ω(0) = 0.

**Figure 9:**
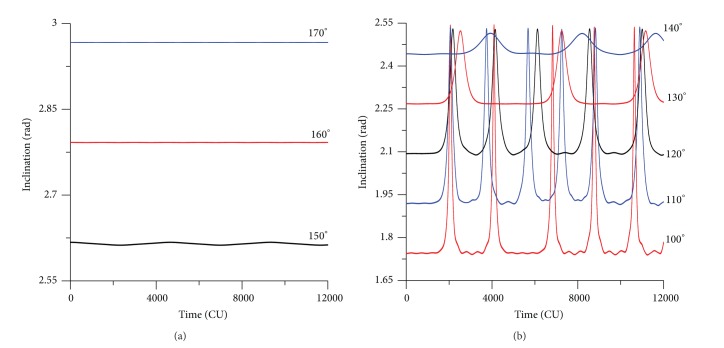
Evolution of the inclination for circular and retrograde orbits (exact model). The initial conditions are *a* = 0.341 canonical units, *e*(0) = 0, *M*_0_(0) = 0, *ω*(0) = 0, and Ω(0) = 0.

**Figure 10:**
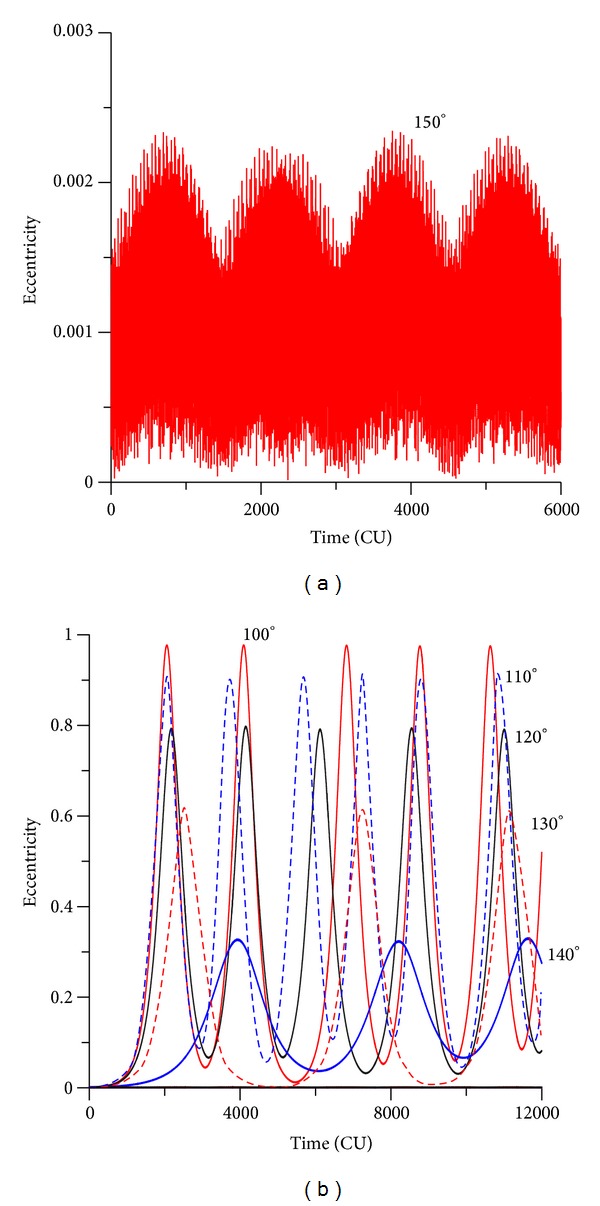
Evolution of the eccentricity for circular and retrograde orbits (exact model). The initial conditions are *a* = 0.341 canonical units, *e*(0) = 0, *M*_0_(0) = 0, *ω*(0) = 0, and Ω(0) = 0.

**Figure 11:**
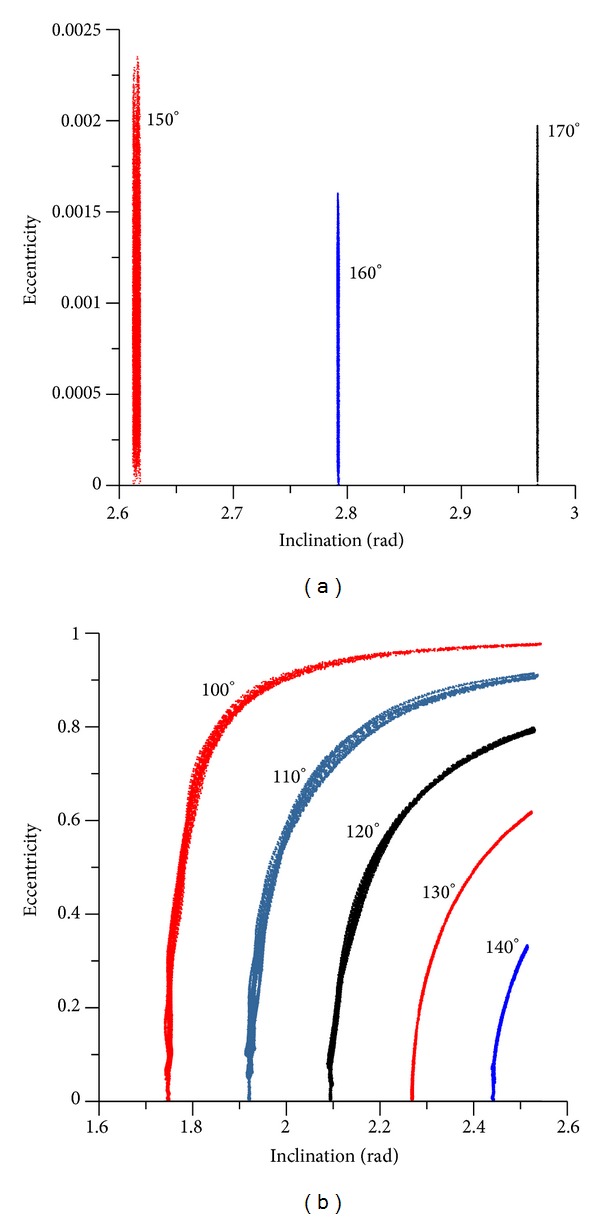
Plot inclination versus eccentricity for circular and retrograde orbits (exact model). The initial conditions are *a* = 0.341 canonical units, *e*(0) = 0, *M*_0_(0) = 0, *ω*(0) = 0, and Ω(0) = 0.

Figure 8(a) shows another view of this typical behavior, emphasizing that for values of the inclination below the critical value there are small changes in the eccentricity, but the inclination remains constant. The cyclic behavior of the inclination and the eccentricity is visible in Figure 8(b). Larger values for the inclination generate small values for the eccentricity, but when the inclination reaches the critical value, the maximum value for the eccentricity is achieved. These same facts are observed for circular and retrograde orbits (Figures 9 and 10). It can be noted that the “U” form of the plots appears again, but this time extra oscillations are present in the lines due to the short periodic terms that are not eliminated. The critical value for the inclination is represented by the complement of the critical inclination. The mirror effect, when compared with the direct orbits, in terms of the inclination and eccentricity, is shown in Figure 11. Larger values for the inclination generate larger values for the eccentricity, but when the inclination reaches the complement of the critical value, the maximum value of the eccentricity is also reached. The use of the complement of the critical inclination shows small variations. The presence of the short periodic terms due to the full model can be seen in the increase of the thickness of the lines, when compared to the averaged models.

## 4. Effects of Varying the Initial Eccentricity

Next, a study is made with the goal of verifying the influence of changing the initial values of the eccentricity in the evolution of the spacecraft for all the models under study.

### 4.1. Averaged Model

Considering the expansions of the disturbing function in polynomials of Legendre until the fourth order in the eccentricity and inclination, we made several simulations varying the initial eccentricity of the orbit while keeping the value of the initial inclination constant. This situation is analyzed for both cases, above and below the value of the critical inclination. 

For orbits with inclination below the critical value and with low values for the eccentricity (almost circular), there are small variations in the evolution of the inclination and eccentricity for both averaged models. The effects of the disturbance of the third body are almost negligible for these types of orbits. As expected, the increase of the initial eccentricity increases the effects of the third body, since the orbit of the spacecraft will have a higher apogee and during this passage the perturbations will have a stronger effect. The perturbation does not affect the stability of the orbits nor produce collisions with the main planet or escape orbits. Another important observation that can be made is regarding the velocity of the oscillations. The increase of the initial eccentricity not only increases the amplitude of oscillations but also reduced the period of the oscillations and so accelerating the dynamics. 

For values above the critical inclination we see that the eccentricity has an inverse behavior, so for higher values of the inclination it reaches minimum values. This analysis confirms that the effect of increasing the initial eccentricity is to accelerate the dynamics of the system, so reducing the period of the oscillations. The main effect of these disturbances is that the orbits that were initially almost circular or slightly elliptical suffer drastic changes and this can cause the collision with the planet or an escape. It is noted that the amplitudes of the variations of the eccentricity are large, even for almost circular orbits, so the third body destroies the stability of the orbits.

### 4.2. Exact Model

Next, the effects of the high eccentric orbits on the orbital elements are studied under the full model, to evaluate the behavior of the inclination and eccentricity without any average and truncation.

**Figure 12:**
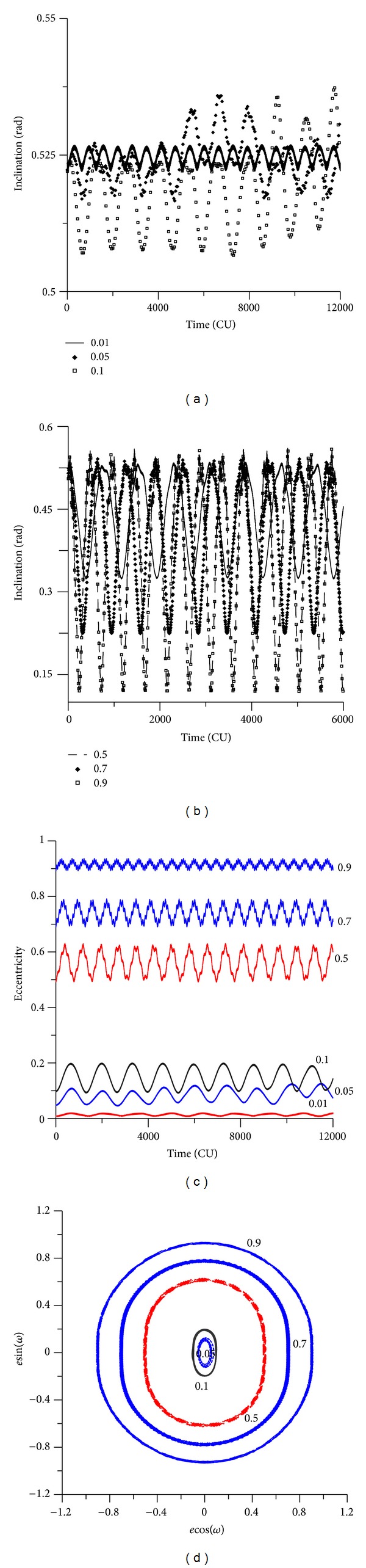
Behavior of orbital elements for initial inclination of 30 degrees. The values of the eccentricity are 0.01, 0.05, 0.10, 0.50, 0.70, and 0.90. The initial conditions are *a* = 0.341 canonical units, *M*_0_(0) = 0, *ω*(0) = 0, and Ω(0) = 0.

Figure 12 shows the evolution of the inclination and eccentricity for different values of the initial eccentricity. For the evolution of the inclination, it is necessary to split Figure 12(a), for eccentricities up to 0.1 and Figure 12(b), for eccentricities from 0.5 to 0.9. Note that the interval of time studied is different, because the increases of the initial eccentricity accelerates the dynamics and so a shorter time, 8000 canonical units instead of 12000, is shown to allow a better view of the results. This fact is visible by the number of peaks of the curves, which increases with the eccentricity. It is clear that the amplitude of the oscillations increases with the initial value of the eccentricity, going from less than 0.003 rad for the situation where the initial eccentricity is 0.01 until more than 0.300 rad for the situation where the initial eccentricity is 0.90. So, the resulting effects of the perturbations can be described by oscillations of the eccentricity with increasing amplitude and a decreasing period when the initial eccentricity of the spacecraft increases. It is, as expected, the same behavior noted when using the averaged models.

**Figure 13:**
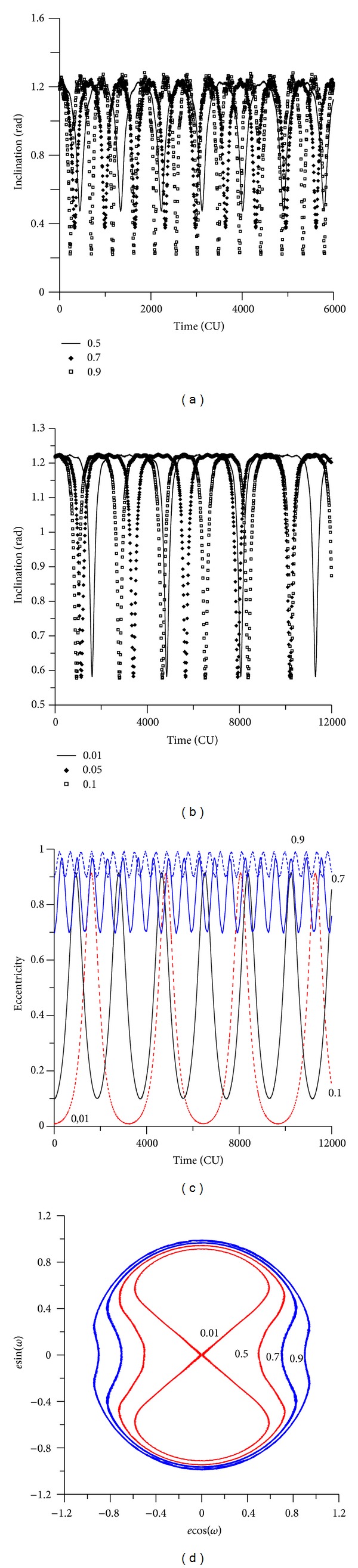
Behavior of the orbital elements for an initial inclination of 70 degrees. The values for the eccentricities are 0.01, 0.05, 0.10, 0.50, 0.70, and 0.90. The initial conditions are *a* = 0.341 canonical units, *M*_0_(0) = 0, *ω*(0) = 0, and Ω(0) = 0.

**Figure 14:**
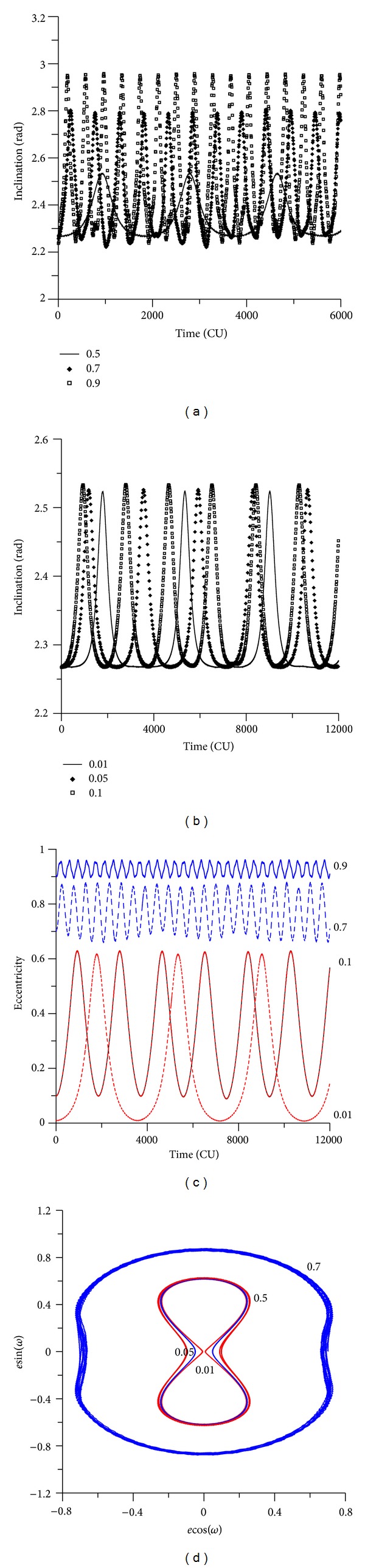
Behavior of the orbital elements for an initial inclination of 130 degrees. The values for the eccentricity are 0.01, 0.05, 0.10, 0.50, 0.70, and 0.90. The initial conditions are *a* = 0.341 canonical units, *M*_0_(0) = 0, *ω*(0) = 0, and Ω(0) = 0.

**Figure 15:**
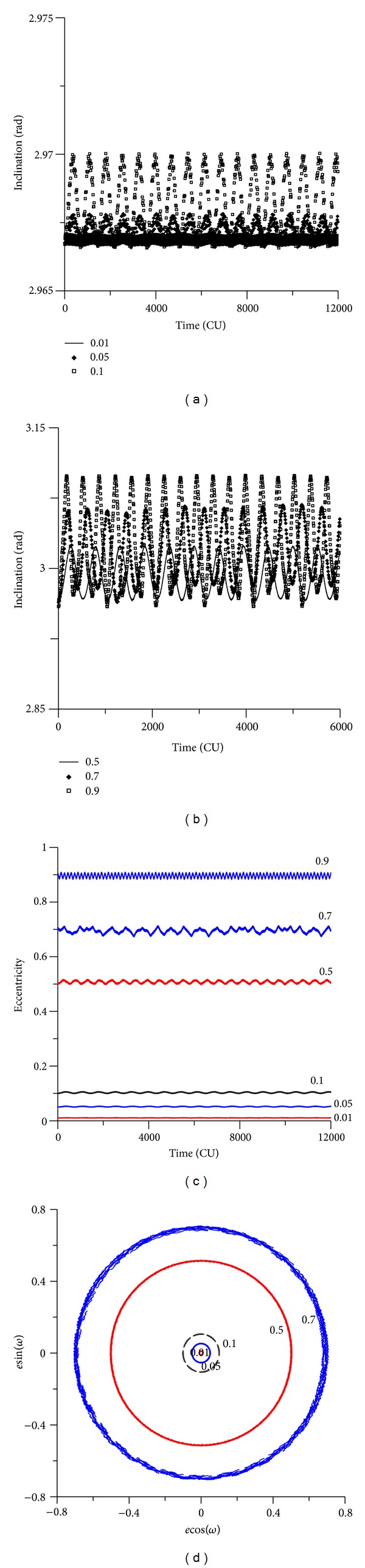
Behavior of the orbital elements for an initial inclination of 170 degrees. The values for the eccentricity are 0.01, 0.05, 0.1, 0.5, 0.7, and 0.9. The initial conditions are *a* = 0.341 canonical units, *M*_0_(0) = 0, *ω*(0) = 0, and Ω(0) = 0.

It is interesting to note that the oscillations in the inclinations occur even for initial inclinations below the critical angle. For example, when the initial inclination is 30 degrees (see Figure 12), for small eccentricities, the inclination has small oscillations, as expected from an initial inclination below the critical value. But, for high eccentricities, the inclination has fast oscillations. For example, for an initial inclination of 0.52 rad (30 degrees) and for the value of the eccentricity of 0.90, the inclination oscillates between 0.14 rad (8 degrees) and 0.52 rad (30 degrees). These angles are below the critical inclination of the third-body perturbation, but the high initial eccentricity caused those oscillations. When the initial inclination is above the critical value, the behavior of the inclination suffers high oscillations (see Figure 13), as expected. For example, for an initial inclination of 70 degrees and high eccentricity (Figure 13(b)), the inclination oscillates between 1.20 rad (70 degrees) and 0.58 rad (34 degrees). Figures 14 and 15 show the results for retrograde orbits, which confirms the results obtained for direct orbits. For all cases (Figures 12, 13, 14, 15(a), and 15(b)), it is visible that the effect of the disturbing body is to increase the eccentricity. 

It is interesting to note that, for values of the initial inclination below the critical values, the eccentricity does not reach larger values, so crashes and escapes do not occur. When the eccentricity becomes larger, the oscillations of the inclination become faster. Figure 12(b) shows a different view of the evolution of the orbital elements, plotting *e*cos(*ω*) against *e*sin(*ω*). It is visible the increase of the thickness of the lines for the regions where the eccentricity has larger oscillations.

The eccentricity keeps the expected behavior. When the initial inclination is below the critical value, the eccentricity shows small oscillations (Figures 12(c) and 15(c)). These oscillations do not affect the orbital stability, and near-circular orbits remain near circular, and orbits with high eccentricity remain with high eccentricity. With an initial inclination above the critical value, the eccentricity suffers several oscillations (Figures 13(c) and 14(c)).

For all the cases where there is an increase in the eccentricity, it oscillates fast. The orbital stability is affected because near-circular orbits are converted into orbits with high eccentricity. The new aspect observed is the acceleration of the dynamics, where the period of oscillation is reduced by the initial eccentricity of the orbit of the spacecraft.

The phase space is also modified by the presence of the high eccentricity and the critical inclination (Figures 12(d), 13(d), 14(d), and 15(d)). Looking at that, it is visible that the high eccentricity destroys the presence of the two equilibrium points. This phase space is invariant when the inclination is below the critical inclination. 

**Figure 16:**
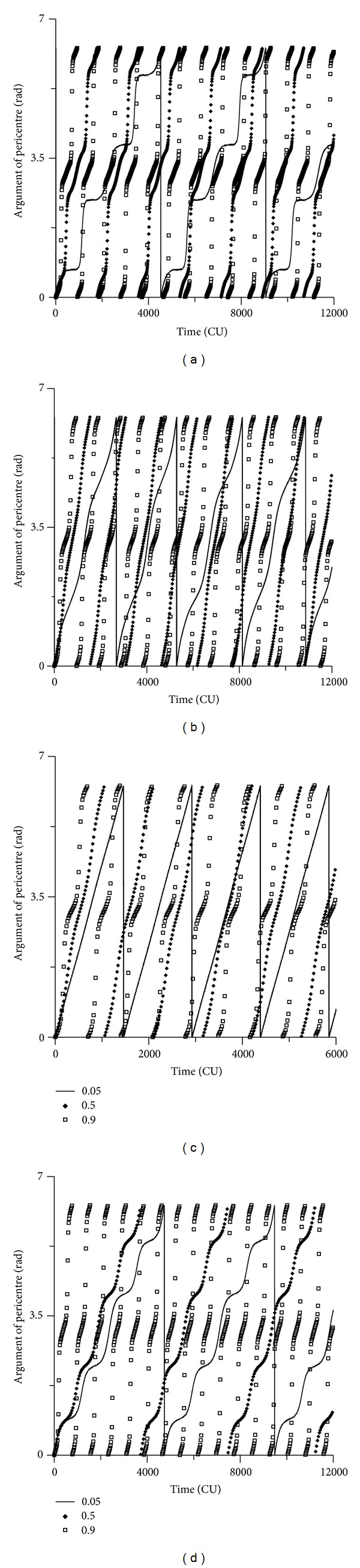
Behavior of the argument of pericenter for initial inclinations of 30 deg, 70 deg, 130 deg, and 170 deg. Initial conditions are *a* = 0.341 canonical units, *M*_0_(0) = 0, *ω*(0) = 0, and Ω(0) = 0.

Next, the evolution of the argument of pericentre is studied. It shows the typical well-known behavior of circulation (Figure 16). The effect of the increase of the initial eccentricity of the spacecraft in the perturbation of the third body is the acceleration of this circulation. When this initial eccentricity increases, the perturbation effect is stronger on the pericentre, and so the oscillations are faster, with shorter period. This fact is noted for all the initial inclinations considered below (Figure 16(a)) and above (Figure 16(b)) the critical values and for retrograde orbits (Figures 16(c) and 16(d)). It is also visible that the velocity of the circulation is faster for orbits below the critical inclination. 

## 5. Conclusions

The present paper has the goal of comparing two averaged methods (single and double) to calculate third-body perturbations against the full restricted three-body problem. The single averaged model is obtained using nonsingular variables, so circular and equatorial orbits can be studied. The initial orbit of the spacecraft is varied, to show its effects in the evolution of the orbital elements.

The effects of the averaged models and its truncations are to produce smoother curves due to the removal of the short periodic terms, as expected, and to accelerate the dynamics by reducing the period of the oscillations. Those facts are quantified in some examples. Both averaged models generate results that are similar to each other and that deviate from the full model when the time advances. It means that the errors are similar in the long term for single and double averaged approximations. The same facts are observed for direct and retrograde orbits.

The increase of the eccentricity of the initial orbit also causes large variations in the inclination, even for initial inclinations below the critical value, which does not happen for initial circular orbits. Equilibrium points are also destroyed in some cases by these high eccentricities. The well-known circulation of the pericenter is also confirmed and the acceleration of its motion for higher initial eccentricities measured.

Regarding computational time, a simulation for 12.000 canonical units of time required about 4.5 minutes for the full model, 21 seconds for the single averaged model, and 12 seconds for the double averaged model.
